# BK channel deacetylation by SIRT1 in dentate gyrus regulates anxiety and response to stress

**DOI:** 10.1038/s42003-018-0088-5

**Published:** 2018-06-28

**Authors:** Diankun Yu, Damek R. Homiack, Edward J. Sawyer, Laura A. Schrader

**Affiliations:** 1 Neuroscience Program, Brain Institute, Tulane University, New Orleans, LA 70118 USA; 2 Cell and Molecular Biology, Tulane University, New Orleans, LA 70118 USA

## Abstract

Previous genomic studies in humans indicate that SIRT1, a nicotinamide adenine dinucleotide (NAD+)-dependent protein deacetylase, is involved in anxiety and depression, but the mechanisms are unclear. We previously showed that SIRT1 is highly activated in the nuclear fraction of the dentate gyrus of the chronically stressed animals and inhibits memory formation and increases anhedonic behavior during chronic stress, but specific functional targets of cytoplasmic SIRT1 are unknown. Here, we demonstrate that SIRT1 activity rapidly modulates intrinsic and synaptic properties of the dentate gyrus granule cells and anxiety behaviors through deacetylation of BK channel α subunits in control animals. Chronic stress decreases BKα channel membrane expression, and SIRT1 activity has no rapid effects on synaptic transmission or intrinsic properties in the chronically stressed animal. These results suggest SIRT1 activity rapidly modulates the physiological function of the dentate gyrus, and this modulation participates in the maladaptive stress response.

## Abstract (web summary)

Diankun Yu et al. show that deacetylase SIRT1 rapidly modulates synaptic properties of the dentate gyrus granule cells and anxiety behaviors through deacetylation of BK channel α subunits. This study provides mechanistic insight into how SIRT1 regulates fight-or-flight stress response.

## Introduction

The sirtuins (SIRT1–7) are a highly-conserved family of nicotinamide adenine dinucleotide (NAD^+^)-dependent protein deacetylases that are expressed in cells throughout an organism, including cells in the nervous system^1,2^. One member of the sirtuin family, SIRT1, the mammalian homolog of yeast Sir2, was first implicated in extension of lifespan by caloric restriction^3^ and has been linked to cell energy metabolism, oxidative stress^4–7^ and neurodegenerative disorders in mammals^8^. Recent studies have implicated SIRT1 in mood disorders in humans. Two independent whole genome sequencing studies, including the Converge consortium^9^, found that SIRT1 single nucleotide polymorphisms are associated with major depressive disorder^10^. In addition, variations in the SIRT1 gene were shown to be associated with risk of anxiety in human population samples^11^.

Animal models of anxiety and chronic variable stress (CVS) are used to determine mechanisms involved in development of anxiety-like and depression-like behaviors. Brain-specific SIRT1 knock-out mice showed reduced anxiety, while SIRT1 overexpressing mice exhibited enhanced anxiety^11^, suggesting that chronic SIRT1 function also modulates anxiety-like behaviors in mice. Further, previous work from our lab showed that SIRT1 is hyper-activated in the nuclear-enriched fraction from the dentate gyrus of the hippocampus of rats exposed to CVS^12^, and chronic inhibition of SIRT1 with sirtinol infused into the dentate gyrus reversed CVS-induced memory impairments and anhedonia^13^. On the other hand, another study demonstrated reduced SIRT1 activity in the hippocampus of chronic ultra-mild stress-treated mice, and increased SIRT1 activation reversed the stress-induced dendritic atrophy and anhedonia in mice^14^. While these results are inconsistent with our previous study, they indicate a critical role for SIRT1 activity modulation during stress, but also highlight the need for further identification of the specific roles of SIRT1 and its substrates in hippocampus function during control and chronic stress conditions. Furthermore, taken together with the human genetic studies, these studies define an important relationship between SIRT1-dependent mechanisms and development of anxiety and depressive disorder-related behaviors.

Interestingly, the roles of SIRT1 in many physiological functions can be opposing and are dependent on the cellular localization of SIRT1 and its substrates. While most studies have demonstrated that SIRT1 is localized to the nucleus and functions in gene transcription^15,16^, recent research shows that SIRT1 is highly expressed in both the nucleus and cytoplasm of neurons^17^ with two nuclear export and import signals within SIRT1’s amino acid sequence^16^, suggesting potential for dynamic changes in localization. The cellular localization and targets of SIRT1 function can change rapidly and likely depend on physiological functional demand, for example, in response to metabolic demand or developmental state. Opposing effects of cytosolic and nuclear SIRT1 have been demonstrated in the fields of tumor growth studies^18^ as well as neuroprotection^19–21^. Furthermore, SIRT1 can affect multiple functions, depending on localization. For example, in pancreatic β cells, cytoplasmic SIRT1 inhibits β-cell survival through Kv1.5 and Kvβ acetylation while nuclear SIRT1 suppression of the expression of uncoupling protein gene 2 increases insulin secretion^22^. Therefore, the localization of SIRT1 and its substrates appears to play a decisive role in the overall functional output of cells. However, the involvement of SIRT1 in anxiety and depressive disorder-related behavior regulation was previously studied under chronic SIRT1 activity modulation such as SIRT1 knockout, overexpression or long-term pharmacological modulation. The implication of rapid effects of cytosolic SIRT1 in CVS and anxiety behavior regulation has not yet been studied.

We hypothesized that rapid deacetylation of targets by cytosolic SIRT1 in the granule cells of the dentate gyrus may mediate differential acute effects compared to chronic modulation of SIRT1 previously described. Thus, dynamic deacetylation of membrane or cytosolic targets may affect neuronal intrinsic properties or synaptic transmission independent of transcription-dependent effects. In this study, we investigated membrane targets of SIRT1 deacetylation that mediate rapid effects in the granule cells of the dentate gyrus in control and CVS animals. Using electrophysiological recordings from granule cells, we found that activation and inhibition of SIRT1 activity rapidly modulated dentate gyrus granule cell intrinsic properties and excitatory transmission. These effects in control mice were mediated through the large conductance, voltage- and Ca^2+^-gated BK channels, and we found that the primary α subunit of BK channels is a direct target of SIRT1 activity. Interestingly, no effect of SIRT1 activity was observed in animals previously exposed to CVS, and chronic stress decreased BK channel membrane expression levels. This mechanism may underlie the stress-induced impairment of SIRT1 rapid effect. Finally, we show that modulation of SIRT1 activity in the dentate gyrus can rapidly affect anxiety behavior in a BK channel-dependent manner.

## Results

To confirm effective manipulation of SIRT1 activity by SIRT1 activator 3 and SIRT1 inhibitor IV in acute brain slices, brain slices were incubated in artificial cerebral spinal fluid (aCSF) with 1:1000 DMSO, 50 μM SIRT1 activator 3, or 1 μM SIRT1 inhibitor IV for 1 h at room temperature, and the acetylation levels of the well-established SIRT1 substrate, acetylated p53 were tested. Our results showed that the acetylation level of p53 was modulated by 50 μΜ SIRT1 activator and 1 μΜ SIRT1 inhibitor IV bath application (supplementary Figure 1), indicating the validation of the activation and inhibition effects of these chemicals.

### SIRT1 activity modulates sEPSC and mEPSC frequencies

**Fig. 1:**
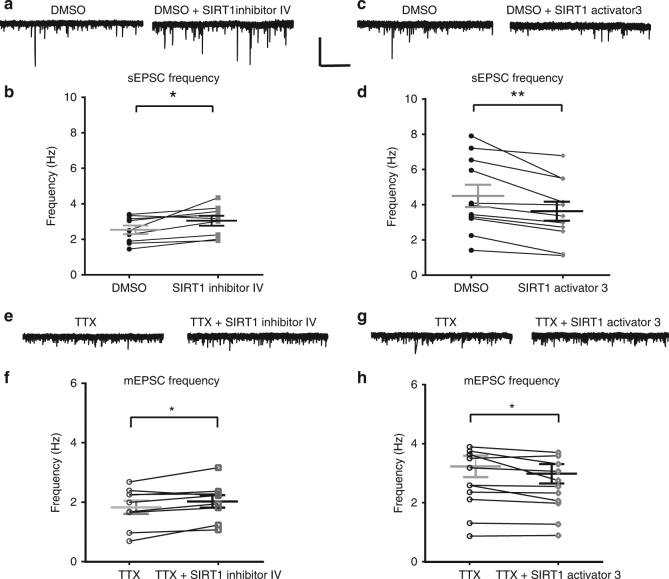
Manipulation of SIRT1 activity affected excitatory synaptic transmission in dentate gyrus from hippocampus slices of control mice. SIRT1 inhibition with SIRT1 inhibitor IV (1 µM) increased the frequency of spontaneous excitatory postsynaptic currents (sEPSCs). **a** Voltage-clamp recording of sEPSCs recorded at −70 mV from the same cell in DMSO and SIRT1 inhibitor IV. **b** Summary graph showing sEPSC frequency from all cells in DMSO and SIRT1 inhibitor IV (*n* = 10). SIRT1 activator 3 (50μM) reduced the frequency of sEPSCs compared to DMSO. **c** Voltage-clamp recording of sEPSCs recorded at −70 mV in the same cell in control (DMSO) and SIRT1 activator 3. **d** Summary plot showing sEPSC frequency in DMSO and SIRT1 activator 3 (*n* = 11). **e** Voltage-clamp recording of miniature EPSCs recorded at −70 mV in the same cell in TTX and SIRT1 inhibitor. **f** Summary plot showing that SIRT1 inhibition (*n* = 9) increased the frequency but not mEPSCs (mEPSCs) compared to TTX. **g** Voltage-clamp recording of mEPSCs recorded at −70 mV in the same cell in control (DMSO) and SIRT1 activator 3. **h** Summary graph showing frequency in DMSO and SIRT1 activator 3 (*n* = 11). SIRT1 activator reduced the frequency of mEPSCs (*P < 0.05, **P < 0.01; paired *t*-test; mean ± SEM shown). Scale bar = 20 pA and 5 s

To determine rapid effects of SIRT1 activation, we first investigated the role of modulation of SIRT1 activity on excitatory synaptic inputs in dentate granule cells. Spontaneous excitatory postsynaptic currents (sEPSCs) were recorded from dentate granule cells in hippocampus slices from control mice before and 10 min after application of 1 µM SIRT1 inhibitor IV or 50 µM SIRT1 activator 3. SIRT1 inhibition had no effect on sEPSC amplitude (*t*_(9)_ = 0.87, *P* = 0.40) but significantly increased sEPSC frequency (*t*_(9)_ = 3.13, *P* = 0.01; Fig. 1a, b). Another SIRT1 inhibitor, sirtinol (30 μM) showed a similar increase of sEPSC frequency (*t*_(5)_ = 2.69, *P* = 0.04; paired *t*-test; supplementary Fig. 2). SIRT1 activation had no effect on sEPSC amplitude (*t*_(10)_ = 1.5, *P* = 0.17) but significantly decreased sEPSC frequency (*t*_(10)_ = 4.14, *P* < 0.01; Fig. 1c, d). Similar effects were observed with another commonly used SIRT1 activator, resveratrol (200 µM), which significantly decreased sEPSC frequency in granule cells (*t*_(8)_ = 4.0, *P* < 0.01; paired *t*-test; supplementary Fig. 2). These rapid effects of SIRT1 manipulation suggest that SIRT1 activity bidirectionally modulated glutamatergic neurotransmission independent of gene transcription.

To determine whether the modulation of sEPSC frequency was action potential-dependent, the effect of SIRT1 activity on miniature EPSC (mEPSC) frequency was investigated by pre-incubation of slices in 1 µM TTX, a voltage-gated sodium channel blocker. Similar to the effect on spontaneous activity, inhibition of SIRT1 activity significantly increased mEPSC frequency (*t*_(8)_ = 2.65, *P* = 0.03; Fig. 1e, f) with no effect on amplitude (*t*_(9)_ = 0.48, *P* = 0.65); while activation significantly reduced mEPSC frequency (*t*_(10)_ = 2.34, *P* = 0.04; Fig. 1g, h) with no effect on amplitude (*t*_(10)_ = 1.67, *P* = 0.13). These results suggest that SIRT1 activity affected glutamate release from the presynaptic terminal, but the smaller magnitude of the change in mEPSC frequency (SIRT1 activator 3: −7.61% mEPSC frequency vs. −19.35% sEPSC frequency; SIRT1 inhibitor IV: +10.65% mEPSC frequency vs. +18.74% sEPSC frequency) suggests that SIRT1 activity also regulated the excitability of presynaptic glutamatergic cells.

### SIRT1 modulates spike width and fast afterhyperpolarization

**Fig. 2:**
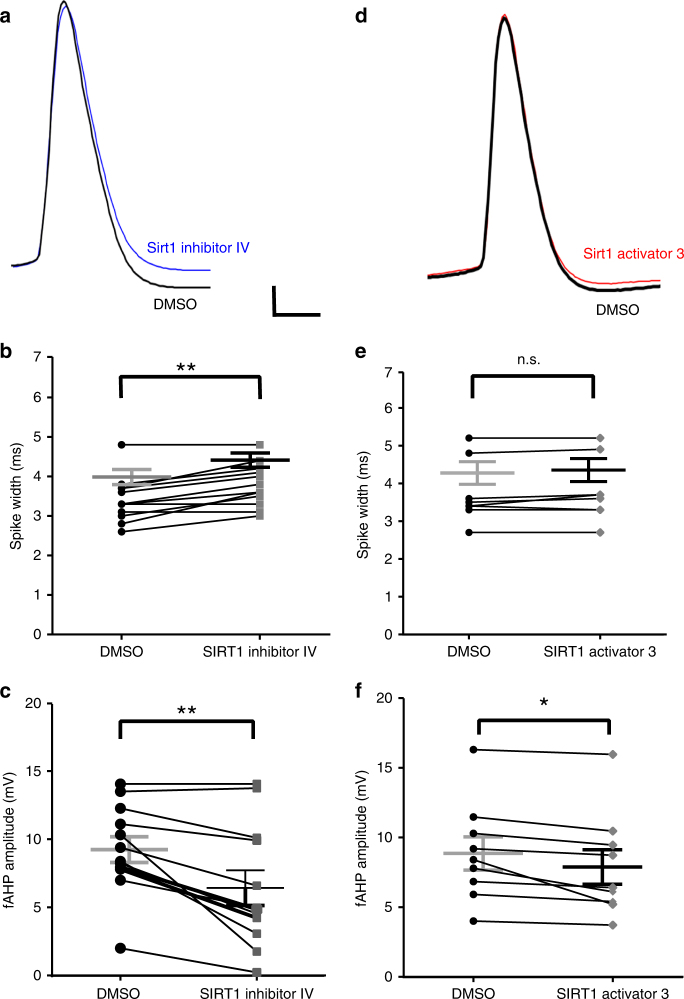
Manipulation of SIRT1 activity affected spike width and fast afterhyperpolarization amplitude in the dentate gyrus granule cells in slices from control mice. SIRT1 inhibition increased spike width and decreased fast afterhyperpolarization amplitude. **a** Example of action potential recorded in control (black) and in SIRT1 inhibitor IV (blue). **b** Summary graphs showing that SIRT1 inhibitor increased spike-width and decreased fast afterhyperpolarization amplitude (**c**) in dentate granule cells (*n* = 12). SIRT1 activation had no effect on spike width but decreased the fast afterhyperpolarization amplitude. **d** Example of action potential recorded in control (black) and SIRT1 activator (red). **e** SIRT1 activator had no effect on spike width, but significantly decreased fast afterhyperpolarization amplitude (**f**, *n* = 9) (*P < 0.05, **P < 0.01; n.s., not significant; paired *t*-test; mean ± SEM shown). Scale bar = 10 mV and 2 ms

We next investigated the rapid effects of SIRT1 activity on granule cell intrinsic properties by investigating changes in the evoked action potentials 15 min after bath application of SIRT1 activator or inhibitor. Manipulation of SIRT1 activity modulated spike width and fast afterhyperpolarization amplitude (Fig. 2). SIRT1 inhibition significantly increased spike width (Fig. 2a, b; *t*_(11)_ = 4.90, *P* < 0.01) and significantly decreased fast afterhyperpolarization amplitude (Fig. 2a, c; *t*_(11)_ = 4.10, *P* < 0.01). On the other hand, SIRT1 activation had no effect on spike width (Fig. 2d, e; *t*_(8)_ = 1.79, *P* = 0.11) but did significantly reduce the fast afterhyperpolarization amplitude (Fig. 2d, f; *t*_(8)_ = 3.10, *P* = 0.02). Input resistance, resting potential, and rise time of action potentials were not affected by SIRT1 inhibition or activation (Supplementary Table 1). These results suggest that SIRT1 rapidly modulated voltage-gated currents that affect the repolarization phase of action potentials in dentate granule cells.

### BK channels mediate the effect of SIRT1 in dentate gyrus

**Fig. 3:**
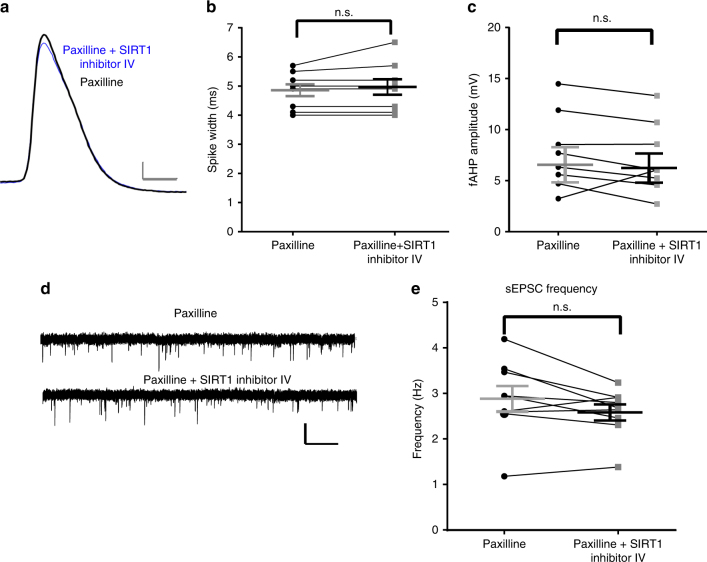
Paxilline pre-incubation blocked the effects of SIRT1 inhibition on spike width and fast afterhyperpolarization amplitude. **a** Preincubation of slices with paxilline to block BK channels blocked the effects of SIRT1 inhibition of spike width and fast afterhyperpolarization amplitude (*n* = 9). The overlapped spike trace before (black) and after (blue) application of SIRT1 inhibitor IV in cells pre-incubated with paxilline. Scale bar = 10 mV and 2 ms. **b** Summary graph showing the effect of SIRT1 inhibitor on spike width and fast afterhyperpolarization amplitude (**c**) in the presence of paxilline. **d** Voltage-clamp recording of sEPSCs in the presence of paxilline and paxilline + SIRT1 inhibitor. Scale bar = 10 pA and 2 s. **e** Summary graph showing preincubation with paxilline blocked the effect of SIRT1 inhibition on sEPSC frequency (*n* = 9) (n.s., not significant; paired *t*-test, mean ± SEM shown)

The decrease in fast afterhyperpolarization amplitude and increase in spike width caused by SIRT1 inhibition, in addition to the effects on glutamate release in response to SIRT1 manipulation, suggested the participation of potassium channels that are critical in the repolarization phase of action potentials, specifically, currents formed by BK channels. To test whether changes in these currents participate in the SIRT1 regulation pathway, acute slices were pre-incubated in aCSF with 5 μM paxilline, a specific blocker of BK channels, and the effects of SIRT1 inhibition on spike width and fast afterhyperpolarization amplitude were measured (Fig. 3). Pre-incubation with paxilline blocked the effects of SIRT1 inhibition, such that SIRT1 inhibition had no further effect on spike width (Fig. 3a, b; *t*_(8)_ = 1.25, *P* = 0.25) or fast afterhyperpolarization amplitude (Fig. 3a, c; *t*_(8)_ = 0.57, *P* = 0.59). This result suggests that BK channels mediate at least part of the effect of SIRT1 activity on spike width and fast afterhyperpolarization amplitude in control animals. In addition, we tested the effect of BK channel blockade on sEPSC frequency. Consistent with the effect of BK channel blockade on spike width and fast afterhyperpolarization, pre-incubation of slices in aCSF with 5 μM paxilline blocked the effect of SIRT1 inhibitor on sEPSC frequency (Fig. 3d, e; *t*_(8)_ = 2.00, *P* = 0.08). These results further confirmed BK channels are a possible regulation target of SIRT1 since the BK channel inhibitor blocked the SIRT1 inhibition effect.

### BK channels α subunits are SIRT1 deacetylation targets

**Fig. 4:**
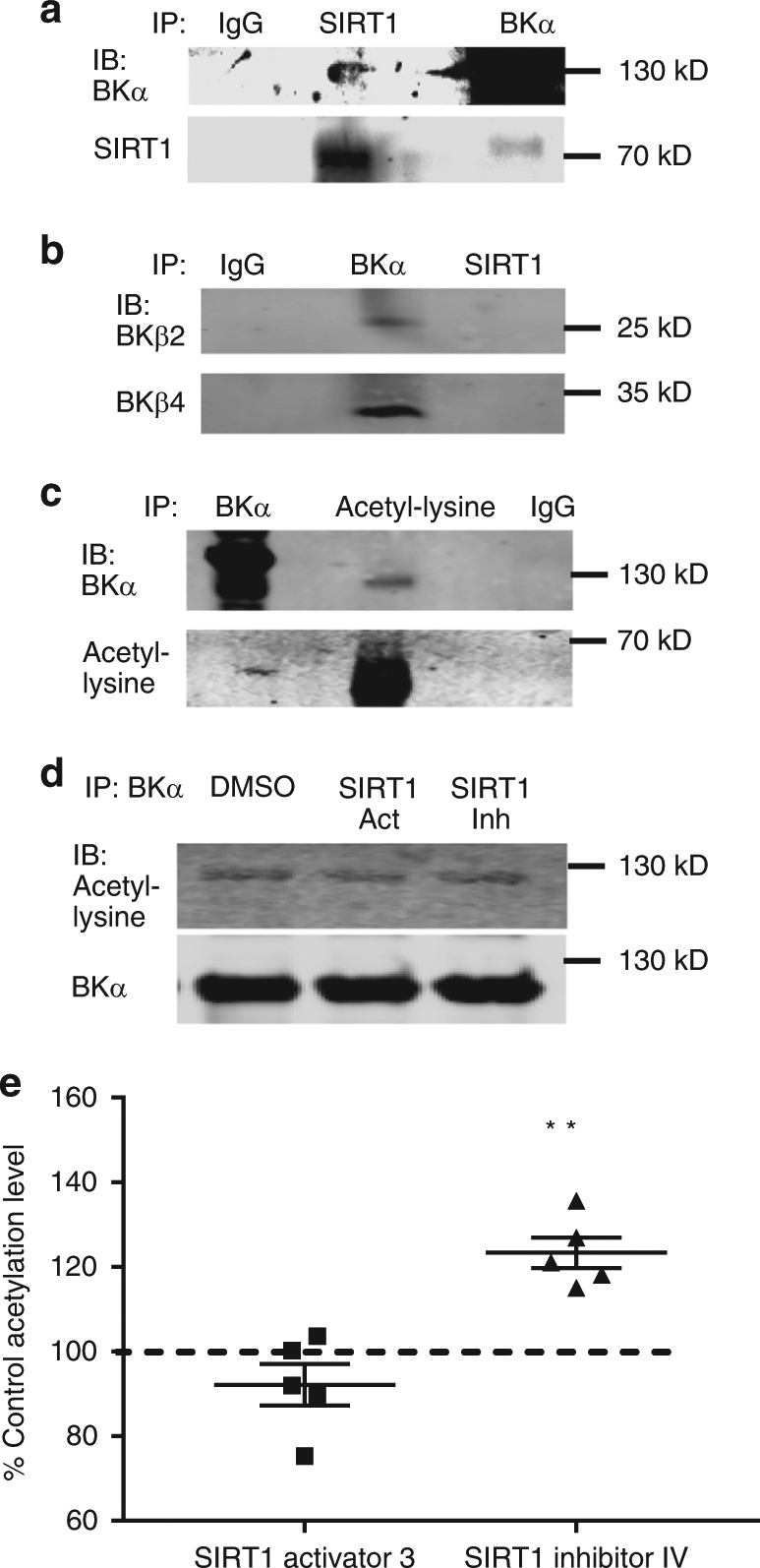
The BK α subunit interacts with SIRT1 and is a target of SIRT1. **a** Immunoprecipitation (IP) with SIRT1 and immunoblot with BK α antibody and IP with BK α and IB with SIRT1 shows that SIRT1 and BK α interact. **b** IP with BK α subunit shows that BK β subunits interacted with the α subunit but not with SIRT1. **c** Co-IP with the BKα subunit and anti-acetylated lysine antibody shows that BK α is acetylated. **d** SIRT1 activation and inhibition in slices and subsequent IP with the BK subunit and immunoblot with the anti-acetylated lysine antibody demonstrated that the BK α subunit is acetylated in hippocampus slices and the acetylation level is regulated by SIRT1 activity. **e** summary bar graph of BK α acetylation showing the percent change from control (DMSO) in the SIRT1 activation and inhibition experiments. SIRT1 inhibition significantly increased BK channel acetylation (**P < 0.01, *n* = 5; post-hoc Dunnett’s multiple comparisons test, mean ± SEM shown). The original blots for each figure can be found in supplementary figure 4

Typically, SIRT1 acts on proteins to remove the acetyl-groups from lysine residues, which may have important regulatory effects on protein–protein interactions or protein function. Since the experiments described suggested that the BK channels are involved in the effects of SIRT1 activity, we hypothesized that the pore-forming BK channel α subunits are direct targets of SIRT1 deacetylation, and that the two proteins directly interact. We investigated protein–protein interactions between BKα and SIRT1 using co-immunoprecipitation (co-IP) of hippocampus tissue. Immunoprecipitation with the anti-SIRT1 antibody and immunoblot with the anti-BKα antibody recognized bands at the ~130 kD level of BKα subunit (Fig. 4a). The co-IP bands indicated a protein-protein interaction between BKα and SIRT1. The reciprocal co-IP experiment of immunoprecipitation with the anti-BKα antibody and immunoblot with the anti-SIRT1 antibody (Fig. 4a) recognized a band at the ~80 kD level and confirmed the interaction. These results show that BKα and SIRT1 proteins interact.

BK α subunits also interact with β subunits which are important for modulation of BK properties^23^. Specifically, β2 and β4 subunits are highly expressed in the hippocampus^24–26^ and are also possible candidates for SIRT1 targets; therefore, we tested whether β2 and β4 subunits may also interact with SIRT1. The BKα and SIRT1 IP samples were immunoblotted with BΚβ2 and BKβ4 antibodies (Fig. 4b). The results showed the BKβ immunoreactivity was recognized in BKα immunoprecipitation sample but not in SIRT1 immunoprecipitation samples, suggesting an interaction with BKα as expected, but no interaction between SIRT1 and BKβ2 or BKβ4.

To further determine if BKα is a SIRT1 deacetylase substrate, we tested whether native BK α subunits have acetylated lysine residues. Proteins were immunoprecipitated from fresh hippocampus lysates with the anti-BKα subunit antibody and immunoblotted with anti-acetylated lysine antibody along with the inverse experiment, proteins were immunoprecipitated with the anti-acetylated antibody and immunoblotted with the BKα antibody (Fig. 4c). The Co-IP bands detected in immunoblotting with both antibodies suggested that the BKα subunit was acetylated in the brain.

Finally, to determine whether SIRT1 deacetylates BKα subunits in the hippocampus, we tested whether the acetylation levels of BKα subunit are modulated by SIRT1 activity (Fig. 4d). We immunoprecipitated hippocampus lysates from acute brain slices that were bathed with vehicle, SIRT1 activator 3 or inhibitor IV for 15 min with BKα antibodies and tested the acetylation level by immunoblotting with anti-acetylated lysine antibodies. Our results showed the percentage change from control in the ratios between acetylated lysine band intensity and BKα band intensity was significantly different (F_(2, 12)_ = 21.04; *P* < 0.001; one-way ANOVA). SIRT1 activation decreased BK channel acetylation (−8 ± 5%, *n* = 5), but the difference was not significant (*P* = 0.24). SIRT1 inhibition significantly increased BK channel acetylation (post-hoc Dunnett’s multiple comparison; *P* < 0.01; 23.35 ± 3.6%, *n* = 5; Fig. 4d, e). These immunoprecipitation and immunoblotting experiments showed that BKα is a potential target of SIRT1 deacetylation. These results suggest this deacetylation of the BKα subunit protein by SIRT1 contributed to the rapid effect of SIRT1 activity on the synaptic activity and intrinsic properties of dentate granule cells.

### SIRT1 modulates BK channel membrane distribution

**Fig. 5:**
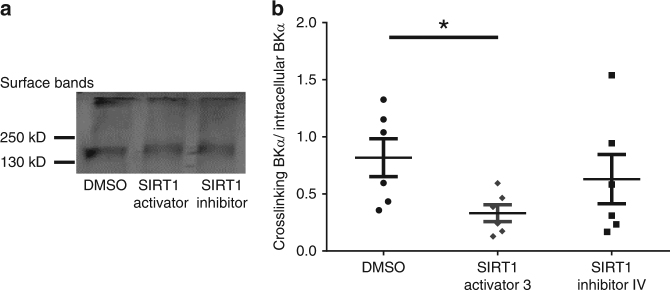
SIRT1 manipulation affected BKα surface expression. **a** BS3 crosslinking of surface proteins shows that SIRT1 activation decreased membrane BKα distribution (surface band). The original blots can be found in supplementary figure 5A. **b** Summary graph showing SIRT1 activation significantly decreased BKα surface expression (Student’s *t*-test, *n* = 6; *P = 0.02)

Internalization of BK channels is important for their physiological functions^27–29^, and protein acetylation has been shown to inhibit protein ubiquitination and trafficking, thus affecting cellular distribution of proteins^30^. Therefore, we hypothesized that deacetylation of BK subunits with SIRT1 will affect BK channel internalization and cellular distribution. To test whether SIRT1 activity rapidly affects BKα surface expression, acute brain slices were incubated with 1:1000 DMSO, 50 μM SIRT1 activator 3, or 1 μM SIRT1 inhibitor IV for 15 min prior to cross-linking all surface proteins in the plasma membranes using BS3, a membrane impermeable amine-to-amine crosslinker. Two bands appeared in our immunoblotting results, the intracellular BKα band around 130kD and the crosslinked membrane BKα band at the top of gels. The ratios between membrane and intracellular BKα immunoreactive bands were determined in these three drug conditions and used to describe BKα subunit internalization and cellular distribution. Our results suggested SIRT1 activation affected BKα subunit surface distribution (Fig. 5, Student’s *t*-test, *t*_(10)_ = 2.67, *P* = 0.02), while SIRT1 inhibition had no effect on internal BKα expression (*P* = 0.50). This suggests that SIRT1 activation decreased the membrane BKα ratios and facilitated BK channels internalization in acute brain slices.

### CVS-treated animals show impaired BKα membrane expression

**Fig. 6:**
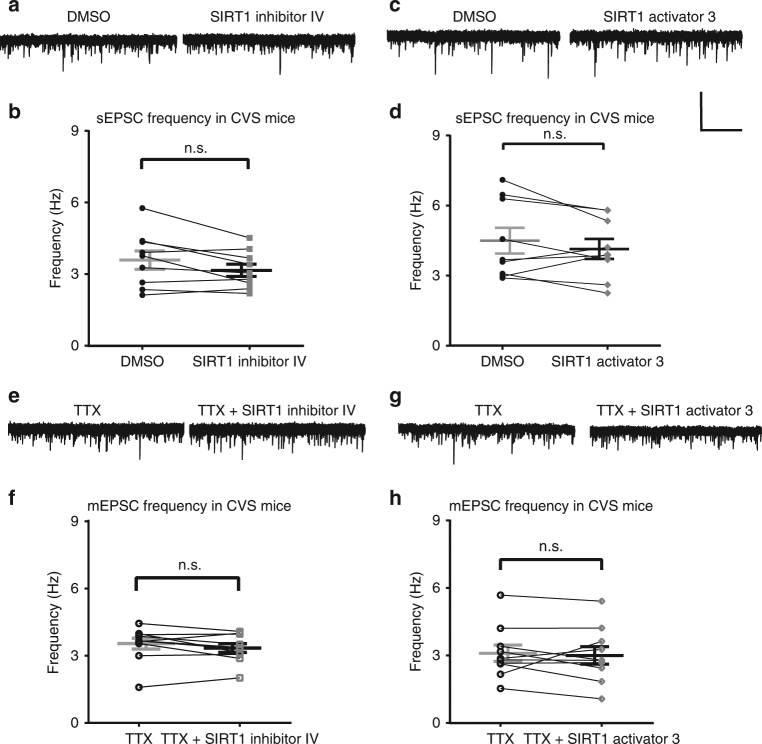
Modulation of SIRT1 activity had no effect on excitatory synaptic transmission in dentate gyrus from hippocampus slices of CVS-treated mice. SIRT1 inhibition with SIRT1 inhibitor IV (1 µM) had no effect on sEPSCs in slices from CVS-treated mice. **a** Voltage-clamp recording of sEPSCs recorded at −70 mV from the same cell in vehicle (DMSO) and SIRT1 inhibitor IV. **b** Summary plot showing sEPSC frequency from all cells in DMSO and SIRT1 inhibitor IV (*n* = 9). Activation of SIRT1 had no effect on the frequency of sEPSCs in slices from CVS-treated mice. **c** Voltage-clamp recording of sEPSCs recorded at −70 mV. **d** Summary plot showing frequency in DMSO and SIRT1 activator (*n* = 9). **e** Voltage-clamp recording of mESPCs recorded at −70 mV in TTX and SIRT1 inhibitor IV. **f** SIRT1 inhibition (*n* = 10) had no significant effect on the frequency of mEPSCs compared to vehicle (DMSO) in slices from CVS-treated mice. **g** Voltage-clamp recording of mESPCs recorded at −70 mV in TTX and SIRT1 activator 3. **h** SIRT1 activation (*n* = 10) had no effect on the frequency of mEPSCs in slices from CVS-treated mice (n.s., not significant, paired *t*-test, mean ± SEM shown). Scale bar = 20 pA and 5 s

Since the previous results from our lab^12,13^ demonstrated increased nuclear SIRT1 activity in the hippocampus of animals exposed to chronic variable stress, we tested the rapid effects of SIRT1 activity in chronic variable stress-treated mice. Stress has been shown to affect glutamatergic transmission in the hippocampus^31–33^, therefore, we hypothesized that SIRT1 activity may also play a role in the stress-induced regulation of glutamatergic activity (Fig. 6). Interestingly, sEPSC and mEPSC frequency in granule cells from slices from CVS-treated mice did not change in response to SIRT1 inhibition (sEPSC: *t*_(8)_ = 2.2, *P* = 0.06, Fig. 6a, b; mEPSC: *t*_(9)_ = 1.53, *P* = 0.16; Fig. 6e, f) or SIRT1 activation (Fig. 6c, d; sEPSC: *t*_(8)_ = 1.28, *P* = 0.24, Fig. 6b; mEPSC: *t*_(9)_ = 0.47, *P* = 0.65, Fig. 6h). The loss of the rapid sensitivity to SIRT1 activity in granule cells from the dentate gyrus of CVS mice compared to control mice, combined with the previously reported elevated SIRT1 activity in the nuclear-enriched fraction of the dentate gyrus after CVS exposure, suggest that chronic stress modulated SIRT1 activity at localized targets, cytoplasmic vs nuclear.

**Fig. 7:**
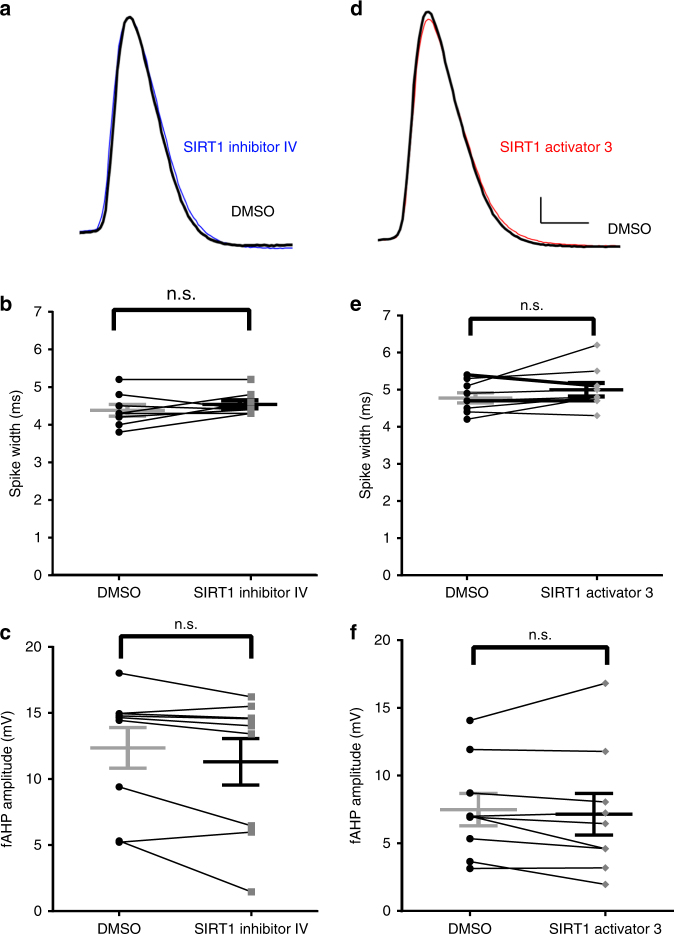
SIRT1 manipulation had no effect on spike width and fast afterhyperpolarization amplitude in the dentate gyrus granule cells in slices from CVS-treated mice. SIRT1 inhibition had no effect on spike width or fast afterhyperpolarization amplitude. **a** Example of action potential recorded in control (black) and in SIRT1 inhibitor (blue). **b** Summary graph showing that SIRT1 inhibitor had no effect on spike width or fast afterhyperpolarization amplitude (**c** *n* = 9) in slices from CVS-treated mice. **d** Example of action potential recorded in control (black) and in SIRT1 activator (red). **e** Summary graph showing that SIRT1 activation had no effect on spike width or fast afterhyperpolarization amplitude (**f**) in slices from CVS-treated mice (*n* = 9) (n.s.,not significant, paired *t*-test, mea*n* ± SEM shown). Scale bar = 10 mV and 2 ms

We also investigated the rapid effect of SIRT1 activity on action potential repolarization phase in CVS-treated mice. Spike width and fast afterhyperpolarization amplitude recorded from granule cells of CVS mice did not change after SIRT1 inhibition (spike width, *t*_(9)_ = 1.71, *P* = 0.13; fast afterhyperpolarization amplitude, *t*_(8)_ = 2.04, *P* = 0.08; Fig. 7a–c) and SIRT1 activation (spike width, *t*_(8)_ = 1.61, *P* = 0.15; fast afterhyperpolarization amplitude, *t*_(8)_ = 0.72, *P* = 0.49, Fig. 7d–f).

**Fig. 8:**
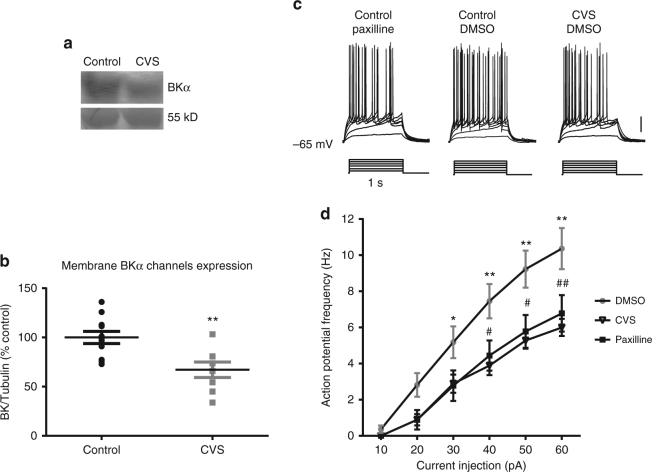
CVS treatment decreased membrane BKα expression and action potential frequency. **a** Western blot showing that BK α subunit expression was decreased in the dentate gyrus of CVS-treated rats compared to controls. **b** Summary plot showing the relative expression of BKα immunoreactivity relative to 55 kD Coomassie band from control and CVS-treated dentate gyrus. The original blots can be found in supplementary figure 5B. **c** Electrophysiological recordings show that CVS decreased the frequency of action potentials evoked by a family of current injection ranging from 10 to 60 pA for 1 s in dentate gyrus granule cells in slices from control animals, and that effect was similar to the frequency of action potentials recorded in the presence of paxilline. **d** Summary plot showing the average ± SEM of frequency in control, CVS-treated mice and paxilline (* or # *p* < 0.05; ** or ## *p* < 0.01; *, ** show post-hoc significance between DMSO and CVS treatment; #, ## show post-hoc significance between DMSO and paxilline treatment. Repeated measures ANOVA and post-hoc Bonferroni’s test)

The electrophysiological results also showed that the spike width in CVS mice (4.6 ± 0.11 ms) had a similar average value compared to that from control mice with paxilline pre-incubation (4.86 ± 0.2 ms) (Fig. 8). Therefore, we hypothesized that the loss of sensitivity to SIRT1 activity in granule cells from stressed animals may be due to the loss of SIRT1-mediated changes in BK currents. The membrane expression of BK channels in control and CVS-treated animals was determined by immunoblotting with anti-BKα antibody in dentate gyrus tissue extract. Membrane BKα immunoreactivity decreased significantly in dentate gyrus extract from CVS-treated rats compared to controls (Fig. 8a, b; Student’s *t*-test, *t*_(17)_ = 3.33, *P* < 0.01).

We investigated the frequency of action potentials evoked by a family of current injections in the dentate gyrus granule cells in hippocampus slices from control mice with DMSO, BK blocker treatment (control mice with paxilline) and CVS treatment (CVS with DMSO) (Fig. 8c). Two-way ANOVA test showed the main effect of current injection and treatment, as well as the interaction between these two main effects were significant (two-way ANOVA, current injection: F_(5,230)_ = 142.30, *P* < 0.0001, treatment: F_(2, 46)_ = 5.78, *P* = 0.006; interaction: F_(10,230)_ = 4.66, *P* < 0.0001). The post-hoc Bonferroni’s test showed action potential frequency in paxilline and CVS treatments are significantly smaller than no treatment (Fig. 8d). This similarity between paxilline and CVS treatment is consistent with the expression data that CVS caused decreased BK membrane expression in the dentate gyrus.

**Fig. 9:**
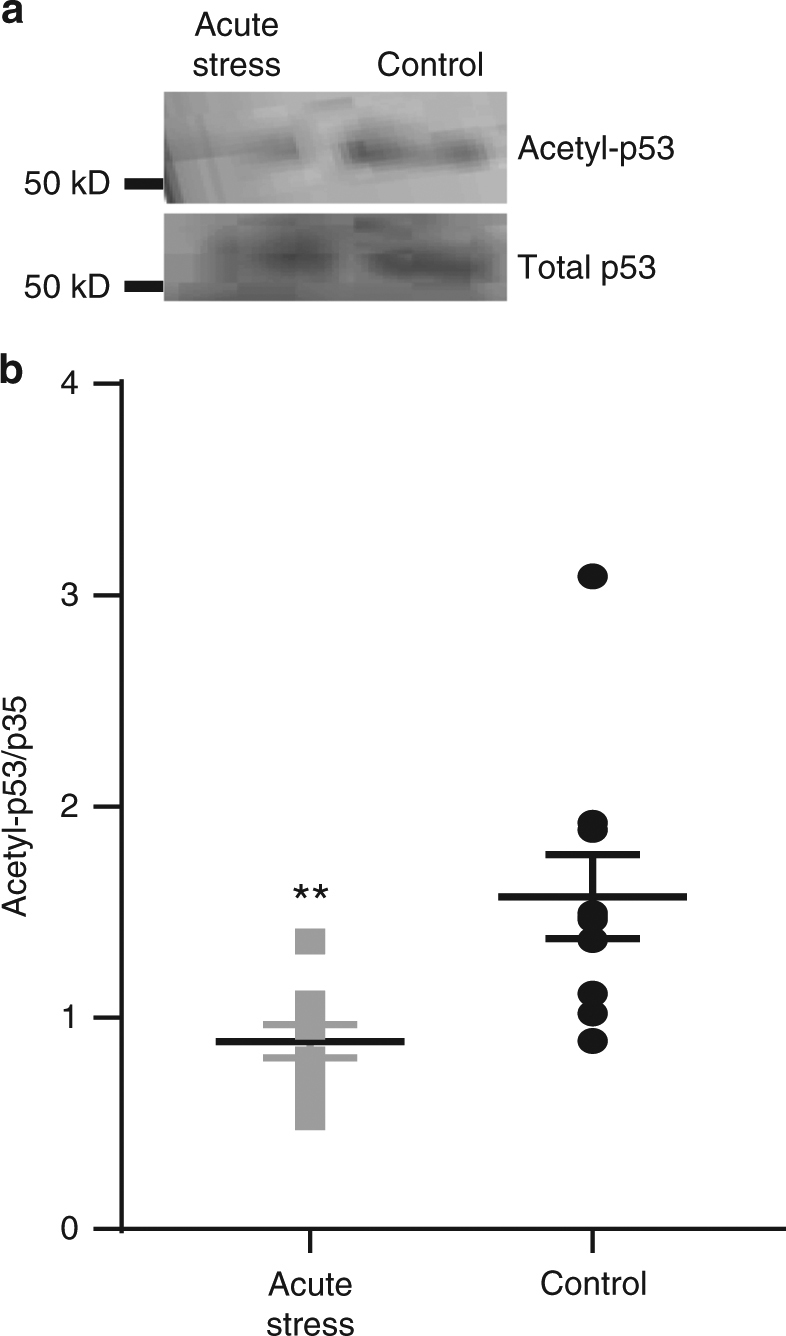
Acute stress increased SIRT1 activation in the dentate gyrus. Animals were exposed to trimethylthiazoline for 30 min as a single acute stressor. **a** Example Western blot showing the levels of acetylated p53 and total p53 in control (*n* = 10) and after acute stress (*n* = 10). **b** The levels of acetylated p53 relative to total p53 measured by immunoblot were significantly decreased by acute stress in the dentate gyrus (**, *p* < 0.01). The original blots can be found in supplementary figure 5C

Since CVS caused an increase in SIRT1 activity in the nuclear fraction, we determined whether an individual acute stressor modulated SIRT1 activation. Our lab previously showed that exposure to the predator odor-mimic, trimethylthiazoline (TMT), activated the hypothalamo-pituitary adrenal axis as measured by circulating corticosterone levels^34,35^, suggesting that trimethylthiazoline-exposure is indeed a stressful condition. Interestingly, single 30 min-stressful trimethylthiazoline exposure caused increased SIRT1 cytosolic activity in the dentate gyrus as demonstrated by decreased acetylation of p53, a SIRT1-specific target (Fig. 9; *t*_(18)_ = 3.2; *P* < 0.01). This result suggests an in vivo mechanism by which SIRT1 can mediate effects following exposure to acute stress. We went on to test the effects of SIRT1 manipulation on anxiety in a mildly stressful paradigm.

### SIRT1 manipulation rapidly affects anxiety behaviors

**Fig. 10:**
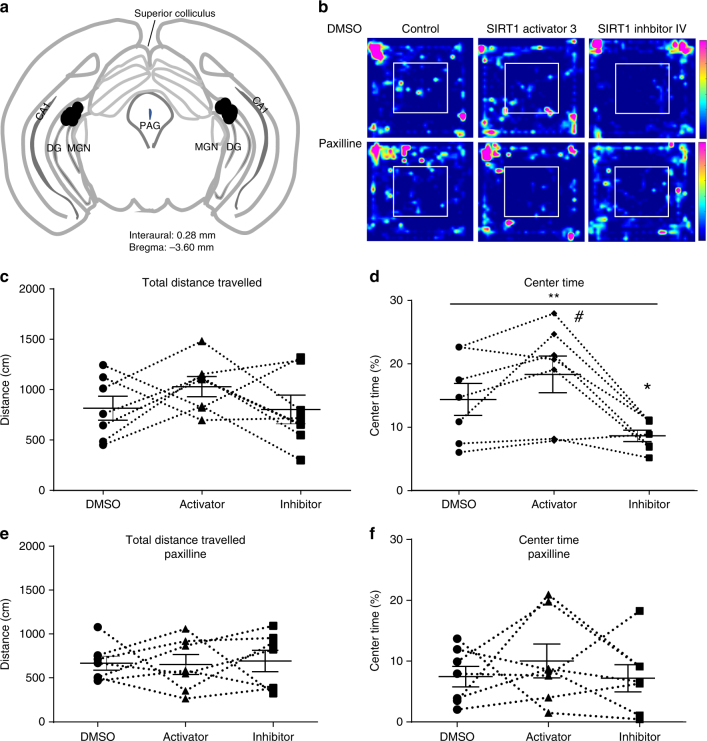
Direct infusion of SIRT1 inhibitor IV and SIRT1 activator 3 into the dentate gyrus rapidly affected anxiety behavior in open field test, and these effects were occluded by BK channel blockade. **a** Schematic diagram showing injection points (black circles) using coordinates from Paxinos and Franklin^58^ atlas to demonstrate placement of infusions into the dentate gyrus. **b** Example heat map of time spent in the field from the same mouse when infused with saline with DMSO, SIRT1 activator or SIRT1 inhibitor (top) and in the presence of paxilline (bottom). **c** Summary plot of distance traveled in the open field in animals infused with saline or SIRT1 activator or inhibitor. **d** Summary plot of percent time spent exploring in the center of the field. The results indicated SIRT1 activity significantly affected the time spent in the center (one-way repeated measures ANOVA, **P < 0.01). SIRT1 activator 3 perfusion increased the time spent in the center (paired *t*-test, #, *P* = 0.07) and SIRT1 inhibitor IV perfusion significantly decreased the time spent in the center of the arena (paired *t*-test, *P < 0.05). **e** Summary plot of distance traveled in the open field in with SIRT1 activation and inhibition in the presence of paxilline to block BK channels. **f** Summary plot of time spent exploring in the center of the open field. Paxilline occluded the effects of SIRT1 activation and inhibition

Previous studies have shown that the dentate gyrus is involved in modulation of anxiety behavior, and signaling in the dentate gyrus is also important for anxiety-like behaviors^36^. To investigate whether the rapid response to SIRT1 activity is important for dentate gyrus function, we infused SIRT1 inhibitor IV or SIRT1 activator 3 bilaterally through pre-implanted cannulas investigated mice behaviors in an open field within 15 min. The positions of the cannulas were confirmed in every mouse after all behavior experiments (Fig. 10a). There were no differences in total distance traveled in the open field between the three groups of mice (Fig. 10b, c, one-way repeated measures ANOVA test, F_(2,12)_ = 1.23, *P* = 0.33). The time spent in the center of the open field was significantly affected by SIRT1 activity (Fig. 10b, d, one-way repeated measures ANOVA test, F_(2,12)_ = 9.94, *P* < 0.01). Mice spend more time in the center of the open field 15 min after SIRT1 activator 3 infusion (paired *t*-test, t(6) = 2.13, *P* = 0.07) and less time after SIRT1 inhibitor infusion (paired *t*-test, t(6) = 2.58, *P* = 0.04) compared to that after the same amount DMSO infusion. These results showed that rapid effects of SIRT1 activity in the dentate gyrus can modulate anxiety behavior in a rapid manner.

We hypothesized that these behavioral effects of SIRT1 manipulation are mediated through its effects on BK channel activity. To test this hypothesis, we blocked BK channels with paxilline to determine if this occluded the effects of SIRT1 manipulation. We co-infused paxilline (5 µM) to block BK channels with the SIRT1 activator and inhibitor. In the presence of paxilline, modulation of SIRT1 activity had no effects on overall activity (Fig. 10e; F_(2, 12)_ = 0.04; *P* = 0.96) or time spent in the center of the open field (Fig. 10f; F_(2, 12)_ = 0.47; *P* = 0.64). These results suggest that rapid effects of SIRT1 activity are indeed mediated through modulation of BK channel activity in the behaving animal.

## Discussion

In this study, we demonstrated that SIRT1 activity rapidly modulated glutamatergic synaptic transmission and intrinsic properties in dentate gyrus granule cells of the hippocampus. SIRT1 inhibition increased the frequency of excitatory synaptic activity and spike width and decreased fast afterhyperpolarization amplitude of dentate gyrus granule cells in hippocampus slices from control mice, while SIRT1 activation decreased the frequency of excitatory synaptic activity and fast afterhyperpolarization amplitude. The increased sEPSC frequency and decreased fast afterhyperpolarization amplitude caused by SIRT1 inhibition in control slices was blocked by the BK channel-specific blocker, paxilline. This occlusion of the SIRT1 effect by BK channel blockade suggested a role for changes in BK channel activity in response to SIRT1 inhibition and BK channel complexes as possible SIRT1 targets. To further investigate the BK channel α subunits as potential targets of SIRT1, we found that SIRT1 interacted with BK channel α subunits but not the β2 or β4 subunits, and the α subunit acetylation levels were modulated by inhibition of SIRT1 activity in hippocampus slices, suggesting direct acetylation of the BK channel α subunit by SIRT1. The lack of effect of SIRT1 activation on channel acetylation suggests two possible explanations. The first is that the BK channel is not highly acetylated at baseline conditions and thus activation of the deacetylase has very little effect. The second is that SIRT1 activation and inhibition and subsequent effects on downstream substrates demonstrate variability in the temporal dynamics. Since SIRT1 activation had a functional effect through modulation of BKα subunit surface expression, the latter possibility seems more likely; however, we can not rule out a possible role of another parallel mechanism in BK channel internalization.

The rapid effects of SIRT1 modulation on excitatory synaptic activity and action potentials in the hippocampus were absent after chronic stress. A possible mechanism for this lack of effect in stressed animals is that the BK α subunit expression was decreased after CVS which resulted in functional changes in cell excitability. These changes were mimicked by the effects of paxilline treatment in slices from control mice, suggesting that CVS decreased the SIRT1/BK channel mechanism of rapid changes in intrinsic excitability. Finally, the behavioral data demonstrated that SIRT1 inhibition in the dentate gyrus of the hippocampus rapidly decreased the time spent in the center of the open field in mice. These effects were occluded by BK channel blockade with paxilline, indicating a similar mechanism as observed in slices preparations. This modulation of the behavioral response in the open field links the rapid effects of changes in SIRT1 activity that we observed in the slice preparation to modulation of anxiety behaviors mediated by changes in SIRT1 activity in the behaving animal. Thus, SIRT1 can facilitate rapid physiological effects independent of transcriptional effects.

The data demonstrating direct de-acetylation of BK channels to modulate functional activity and surface expression is consistent with the role of protein acetylation as a mechanism of protection from ubiquitination to stabilize proteins. Ubiquitination occurs at lysine sites within the protein amino acid sequence, and acetylation at those sites competitively inhibits ubiquitination and subsequent protein degradation. Thus, dynamic modulation of acetylation and deacetylation is important for regulation of cell functions in various contexts^30,37–39^. For example, our results are consistent with the previous study that the deacetylation of a type of peripheral ion channels, epithelial Na^+^ channels (ENaC), increased their ubiquitination and decreased membrane expression^40^. Furthermore, our results are also consistent with several studies indicating the important deacetylation function of cytosolic SIRT1 in the general principal of changes in cell and synapse structure. SIRT1 deacetylation of cytoskeleton-associated proteins is an important factor for cell structure and movement such as tubulin^41^. Cortactin is acetylated by SIRT1^42^ and this dynamic acetylation plays a role in synapse structure and PSD 95 localization^43^. Our research confirmed the deacetylation function of the cytosolic SIRT1 and extends the SIRT1 deacetylation target to ion channels in the brain. Since BK channels are expressed in many neuron types, the deacetylation of BK channels could be an important contributor to synapse structure and function. In addition, BK channels are expressed in cells throughout the body, our data provide another mechanism through which SIRT1 can modulate cell function, which may be used in cells throughout the body. While we did not investigate acetylation sites within the BK amino acid sequence in this study, several candidate sites exist, and the effects of local SIRT1 on acetylation and ubiquitination of these sites within the BK sequence will be investigated in future studies.

Since the dentate gyrus is implicated in regulation of anxiety and stress behaviors^44,45^, the present study focused on the role of SIRT1 in modulation of cell excitability and synaptic inputs of granule cells of the dentate gyrus. Interestingly, a recent study showed that inhibition of the principal neurons of the dentate gyrus or CA3 can suppress anxiety^46^. Consistent with this study, our electrophysiological and behavioral results demonstrated that SIRT1 activation can rapidly decrease glutamatergic excitatory synaptic transmission in the dentate gyrus and has anxiolytic effects. These effects were detected within 10–20 min after pharmacological manipulation of SIRT1 activity and were blocked by blockade of BK channels, indicating they were independent of gene transcription and likely mediated by cytosolic SIRT1-mediated effects on BK channels.

Our findings support and provide more details for the theory that SIRT1 links energy metabolism and mood disorder regulation^47^. SIRT1 activation is NAD+-dependent, and NAD+ is a cofactor that is important in glycolysis and the Kreb’s cycle, key metabolic pathways important to ultimately produce adenosine triphosphate. Depletion of NAD+ can inhibit glycolysis^48^, and thus consumption of NAD+ by SIRT1 activity can modulate gluconeogenic and glycolytic pathways^49^. Interestingly, we also showed that cytosolic SIRT1 activity in dentate gyrus is increased following acute stress, a period of high metabolic activity, which likely enables the important role of SIRT1 rapid effects in responding to acute stress. It is expected that increased SIRT1 activity induced by acute stress would decrease glutamatergic transmission in the dentate gyrus and modulate anxiety behavior, functioning as an adaptive role after an acute stress. Indeed, our behavior results showed SIRT1 manipulation affects anxiety behavior during a mild stressor. Specifically, our results showed that exogenous inhibition of SIRT1 during open field exploration, a mild stressor, increased anxiogenic behavior, an effect that was blocked by paxilline, suggesting it is mediated through SIRT1’s effects on BK channel activity.

The present study, along with our previous research that showed increased nuclear SIRT1 activity after CVS^12,13^, indicate SIRT1 activity and cellular localization in the dentate gyrus is dependent upon stress conditions, and importantly SIRT1 activity rapidly modulated mouse anxiety behaviors. Taken together, the SIRT1 cytosolic activity increase induced by the metabolic state change in a single stressor may initially contribute to changes in the synaptic inputs and intrinsic properties of dentate gyrus granule cells which suppress stress-induced anxiety to respond effectively to an acute stressor. These results suggest that levels of SIRT1 activity in the dentate gyrus dynamically modulates the passive (higher anxiety) vs. active (less anxiety) responses to an acute stressor^50^ that would be determined by different environmental conditions. Interestingly, loss of the ability of SIRT1 to affect the physiological conditions of dentate gyrus granule cells during chronic stress may contribute to the maladaptive processes that occur in response to repeated stressors and increased anxiety associated with responses to chronic stress.

Our results show that SIRT1/BK pathway is a candidate mechanism for adaptive processes that occur in response to acute stress. Within the brain, ATP is primarily consumed by electrical signaling processes, including synaptic transmission and action potential generation^51^. Increased cytosolic SIRT1 activity as demonstrated in our study, likely results in enhanced NAD+ consumption, and decreased sEPSC frequency in control mice. Since glutamatergic transmission is metabolically highly demanding, the reduction in sEPSC frequency may be an adaptive mechanism to compensate for increased NAD+ consumption in control mice. However, prolonged or repetitive stressors may cause long-term reductions in NAD+ levels, thus preventing SIRT1 activation. Reduced NAD+ levels are also indicated in various pathological conditions, including physiological conditions such as aging^52^ and further limits activity of cytosolic SIRT1 pathway. In this way, the adaptive loss of SIRT1/BK pathway and the concomitant loss of metabolic adaptation may contribute to the formation of depressive disorders under prolonged stress or repeated stressors.

## Methods

### Animals

All animals and procedures were approved by Tulane Institutional Animal Care and Use Committee (IACUC) according to National Institutes of Health (NIH) guidelines. Male mice were used at 4–10 weeks old C57 male mice ordered from Charles River. All mice were housed on a 12:12 light/dark cycle and received food and water ad libitum. Animals were handled daily to avoid acute stress prior to experiments. Mice were group housed except for CVS-treated mice that were single-housed according to CVS paradigm. Rats were used for some CVS and acute stress experiments. Male rats (56 days old) were ordered from Envigo. Animals were pair-housed and adapted to the vivarium for 1 week prior to experiments.

### Electrophysiology

Hippocampus slices were prepared according to previously described methods^53^. Briefly, mice were deeply anesthetized with isoflurane and decapitated, and the brains were rapidly removed. Acute hippocampus brain slices were prepared in ice-cold oxygenated (95% O_2_/5% CO_2_) cutting artificial cerebrospinal fluid (aCSF) with a vibratome. Slices were incubated at 35 °C for 30 min then held at room temperature in oxygenated standard aCSF until use. In some experiments, 1 μM tetrodotoxin (TTX, Alomone Labs Cat #T-550) or 50 μΜ paxilline (Sigma Aldrich, Cat #57186-25-1) was applied during the incubation to inhibit voltage-gated sodium or large conductance calcium-dependent potassium channels (BK channels) respectively. Dentate gyrus granule cells were identified under infrared differential interference contrast (DIC) videomicroscopy and only cells from the upper blade of dentate gyrus were targeted for whole-cell patch clamp recordings. Recordings were done at room temperature using a Digidata 1322 A digitizer with Multiclamp 700B amplifier. Series resistance was monitored throughout the recording, and any cells with access resistance more than 20 MΩ or more than 20% change were excluded from further analysis. sEPSC and mEPSC events were recorded at holding potential of −70 mV under voltage clamp with pClamp software and analyzed by Minianalysis. Action potentials were evoked by small current injection ranging from 10 to 60 pA from a holding potential of −65 mV under current clamp. Fast afterhyperpolarization amplitude was defined as the voltage difference between fast afterhyperpolarization and threshold of the first evoked spike at 50pA current injection, and the spike width was defined as the time difference between up- and down-stroke at the threshold voltage. Statistical analysis was done in Graphpad Prism 6. Unless otherwise specified, paired t-tests were used to determine significance between control and drug application. Solution Recipe**:** Cutting aCSF (mM): 125 NaCl, 2.5 KCl, 26 NaHCO_3_, 1.24 NaH_2_PO_3_, 25 Dextrose, 8 MgSO_4_, 0.25 CaCl_2_; Standard aCSF (mM): 125 NaCl, 2.5 KCl, 26 NaHCO_3_, 1.24 NaH_2_PO_3_, 25 Dextrose, 2 MgSO_4_, 2 CaCl_2_; Pipette solution (mM): 120 Kglu, 20 KCl, 0.2 EGTA, 10 Hepes, 4 NaCl, 4 Mg^2+^ATP, 14 phosphocreatine, 0.3 Tris GTP. pH was adjusted to 7.2–7.25 by KOH. Osmolarity was adjusted to 305–315 mOsm by sorbitol. The main SIRT1 inhibitor and activator drugs were made as 1000× stock solution in DMSO stored aliquoted at −20 ℃, and bath applied at a concentration demonstrated before^54,55^ prior to bath application: SIRT1 inhibitor IV (Calbiochem Cat #566325): 1 µM; SIRT1 activator 3 (Santa Cruz Biotechology, Inc. sc-222315): 50 µM.

### Chronic variable stress

CVS was conducted using a similar method as previously reported^12,13^. Briefly, the CVS paradigm for mice consisted of randomized stressors twice a day daily for 21 days, with occasional overnight stressors. Morning and afternoon stressors consisted of cold swim (8 min at 20 °C tap water), cold room (1 h at 4 °C), warm swim (20 min at 35 °C tap water), shaking plate (1 h on shaking plate), wet bedding (1 h). Overnight stressors consisted of no bedding, single housing and overcrowding (6 mice in each cage). Daily food consumption and weight gain were monitored and demonstrated the CVS mice were stressed as CVS decreased daily food consumption and weight gain (Supplementary Fig. 3). Mice received 21 days of stressors that concluded the day before recordings to avoid the confounds of acute stress. For recordings, all control and CVS mice were handled the same way and sacrificed at the same time of the day (11 am ± 30 min).

Rats were used for some CVS experiments. Rats were divided randomly into 2 groups, control and CVS. CVS stressors for rats consisted of 2 stressors a day for 14 days and included warm swim (20 min at 31–33 °C), cold swim (10 min at 16–18 °C), cold room (1 h at 4 °C, two rats per cage without bedding), rotation (1 h at 100 rpm), social isolation (overnight, one rat per cage), and social crowding (overnight, 6 rats per cage). Overnight stressors (social isolation or social crowding) began immediately after cessation of the afternoon stressor and concluded with the start of the next day’s morning stressor. All animals were weighed every other day. On the morning of the 15^th^ day, approximately 12 h after the last stressor, animals were killed by rapid decapitation and trunk blood collected. The hippocampus was extracted and subdivided into CA1, CA3 and dentate gyrus regions for protein extraction. Animals were sacrificed between 0900 and 1000, when corticosterone levels are lowest and at least 16 h after administration after the last stressor.

### Acute stress

Rats used in acute stress experiments were obtained and housed as described above. For experiments that investigated the effects acute stress in rats, rats were stressed as previously described^34^. Briefly, we used the predatory odor 2,5-dihydro-2,4,5-trimethyl thiazoline (TMT), an innate stressor in rodents which has been shown to elicit strong activation of the HPA axis and an acute increase in serum corticosterone levels^56,57^. Prior to exposure to TMT, all animals were removed from the vivarium and experimental and non-experimental animals were placed in separate isolated rooms to prevent unintentional scent exposure to non-experimental animals. Animals were given 2 h to acclimate prior to TMT exposure. For experimental groups, a small Petri dish with filter paper soaked with 150 µL of undiluted TMT (PheroTech Inc., Canada) was placed in the center of the animal’s home cage. Rats were exposed to TMT for 30 min. After the 30 min, animals were immediately killed by rapid decapitation, trunk blood collected, and adrenal glands weighed. The hippocampus was extracted and subdivided into CA1, CA3, and dentate gyrus regions for protein extraction.

### Immunoprecipitation/Co-immunoprecipitation (IP/Co-IP)

Hippocampus tissue was collected from acute mouse brain slices which were prepared in the same way as those in electrophysiological experiments. The brain slices were incubated in either 1:1000 DMSO, 50 μM SIRT1 activator 3 or 1 μM SIRT1 inhibitor IV before IP procedures. Pierce^TM^ Classic Magnetic IP/Co-IP Kit (Thermo Scientific, 88804) was used for IP/Co-IP experiments. To avoid post-translational modification of proteins, phosphatase inhibitor cocktail (Calbiochem, Cat.# 524627, 1:100), 10 mM sodium fluoride, 20 mM sodium butyrate and 1 mM sodium orthovanadate as well as protease inhibitor (Calbiochem, Cat.# 535142, 1:100) were added to the NP-40 lysis buffer provided in the kit. The protein concentration was determined, and all samples were diluted to the same concentration before IP. The immunocomplex was 2 μg antibodies in 200 μL lysis buffers with 0.6 mg/mL proteins. All procedures followed the instruction of Pierce^TM^ Classic Magnetic IP/Co-IP Kit. The Western blot analysis protocols were followed to immuno-blot (IB) our target protein.

### Bis(sulfosuccinimidyl) suberate (BS3) crosslinking

Hippocampus and surrounding cortical slices were randomly divided into 3 groups and incubated in 1:1000 DMSO, 1 μM SIRT1 inhibitor IV, or 50 μM SIRT1 activator 3 for 15 min. After wash, slices were placed into ice-cold standard aCSF containing 2 mM bis(sulfosuccinimidyl) suberate (BS3) (ThermoFisher, 21580) for crosslinking for 30 min. The reaction was quenched with 100 mM glycine. Slices were washed with ice-cold standard aCSF. Hippocampi were removed from slices and lysed in the lysis buffer with protease inhibitor and 20 mM sodium butyrate, aliquoted and processed with the same western blot analysis protocol, except that proteins were transferred to the membrane overnight at 40 mV in a 4 °C cold room.

### Western blot analysis

Tissue from rats exposed to CVS or controls was gathered by bilaterally sub-dissecting the hippocampus into dentate gyrus, CA3, and CA1 sub-regions as previously described before^12^. Dentate gyrus tissue was sub-fractionated using a commercially available kit (Millipore compartmental fractionation kit, 2145) per manufacturer’s instructions to generate a membrane enriched fraction. For mouse tissue, the whole hippocampi were collected from acute brain slices prepared in the same way as electrophysiological experiments and lysed with NP-40 lysis buffer with protease inhibitor and 20 mM sodium butyrate. The protein concentration was determined by Bio-Rad DC protein assay (BioRad, Hercules, CA, Cat #500) and all samples in the same experiments were diluted to the same concentration before further procedure such as IP or aliquot. To denature and reduce the samples, sample buffer (5×, Thermo-Fisher Scientific, 39001) and dithiothreitol (DTT, final concentration to 50 mM) were added to all samples and samples were boiled for 5 min before loading. The same amount of protein was loaded into 10% SDS-PAGE gels, separated and transferred to a PVDF membrane. The membranes were blocked in Odyssey^®^ Blocking Buffer (TBS) (LI-COR_®_, Part No. 927-50000) for 1 h at room temperature with constant rocking. The membranes were incubated with primary antibodies including anti-BK α (Neuromab, L6/60, 1:1000), anti-BK β2 (Neuromab, N53/32 1:1000), anti-BK β4 (Neuromab, L18A.3, 1:1000), anti-SIRT1 (Santa Cruz, sc-15404, 1:1000), anti-acetylated lysine (Cell Signaling, 9441 S, 1:1000) or anti-GAPDH (Cell Signaling, 1:10,000) primary antibodies at 4 °C overnight with constant rocking. The membranes were washed three times with TTBS and incubated with fluorescence conjugated secondary antibodies (IRDye^®^ 680RD Goat anti-mouse, IRDye^®^ 800CW Goat anti-rabbit, 1:10,000) for 1 h at room temperature. Immuno-reactive bands were acquired using Odyssey^®^ imaging system (LI-COR). Results were analyzed by Image Studio Lite V5.2.

### Surgery and behavior

Animals were anesthetized by ketamine/xylazine and maintained on isoflurane and placed in a Kopf stereotaxic instrument with the tooth bar set level with the interaural 10 line. Two 2.5 mm-long stainless steel 23-gauge cannula were implanted bilaterally above the dentate gyrus of the hippocampus, coordinates: AP - 3.6 (Bregma), L ± 2.2 ± 0.1, DV - 3.0 (skull). The open-field behavior experiment was conducted two weeks after surgery in a repeated-measures design in dim red light at dark cycles. All mice received three different drug conditions: 1:1000 DMSO, 1 μM SIRT1 inhibitor IV and 50 μM SIRT1 activator 3 in sterile saline with or without 5 μΜ paxilline and the sequence of three drug application was randomized with one-week interval between any two conditions to avoid interaction between conditions. Mice were handled every day by the experimenter to reduce stress during experimentation. Before every experiment day, the mice were habituated in the experiment room for 2 h every day for 3 days and the infusion posture was mimicked for 2 min to decrease the discomfort caused by handling and drug infusion. On the experiment day, drugs were injected at a flow rate of 100 nL/min through the bilateral cannula (PlasticsOne 1 mm C315GA 33GA internal cannula) pumped by Osmotic pump and two 250 µL infusion syringe (Hamilton) for 2 min. Fifteen minutes after drug infusion, the 5-min open field test was conducted. Infrared beams and computer-based software Fusion was used to track mice and calculate mice activity and time spent in the center (8 in × 8 in) of open field (16 in x 16 in), defined as entire body (4 paws) in the center. Matlab R2017b was used to calculate the heat map shown in Fig. 10.

Mice were transcardially perfused 1.5 h after last behavior tests and mice brain were collected for immunohistochemistry and cannula guides position confirmation.

### Data availability

Data reported here are available within the manuscript or upon reasonable request to the corresponding author.

## Electronic supplementary material

Supplemental material
