# Supplementary material for: BK channel deacetylation by SIRT1 in dentate gyrus regulates anxiety and response to stress
Source: Commun Biol. 2018 Jun 28;1:82. doi: 10.1038/s42003-018-0088-5 (PMC6123630; doi:10.1038/s42003-018-0088-5)
Supplement: Supplementary file 1 — Supplemental material [file 42003_2018_88_MOESM1_ESM.pdf]

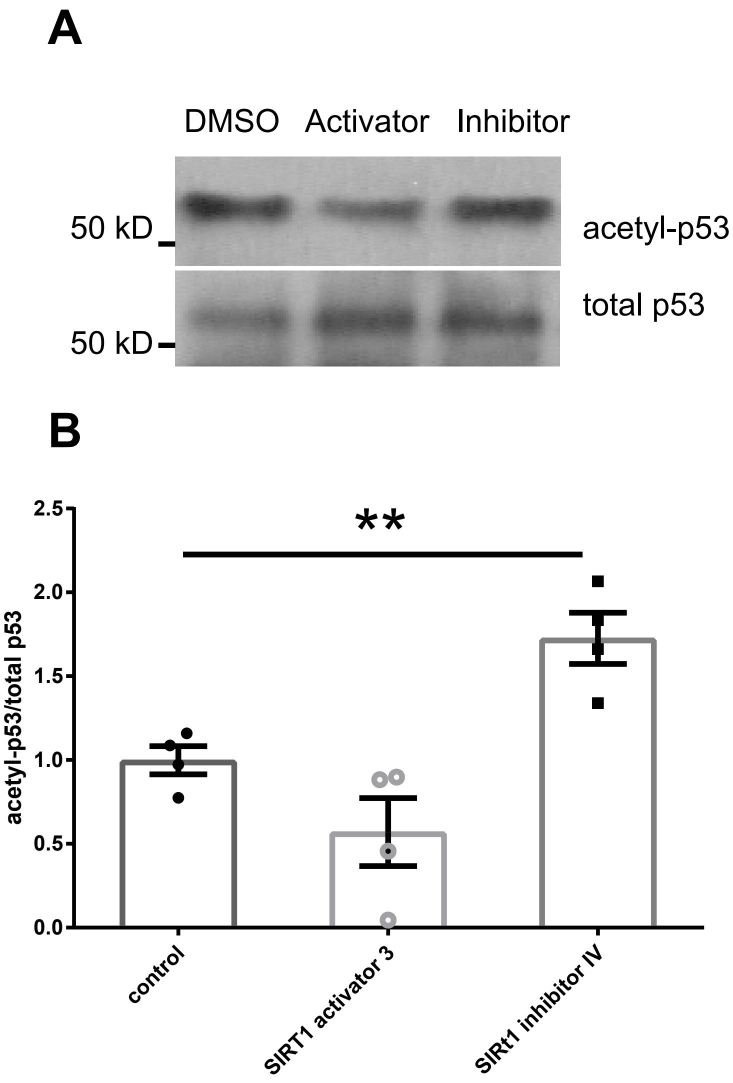

Supplementary Figure 1. SIRT1 activator 3 and SIRT1 inhibitor IV modulated the acetylation levels of p53. We tested whether our SIRT1 activator IV and activator 3 could modulate protein acetylation level in our acute brain slices using the ratio between acetylated p53 and p53. A. Western blot imaging showing acetylated (upper) and total (lower) p53. B. Summary graph showing the results of 4 separate experiments. One way ANOVA showed acetylation level of p53 is significantly regulated by SIRT1 drugs (one-way ANOVA, $F_{(2,9)}$ =14.26, P<0.01).

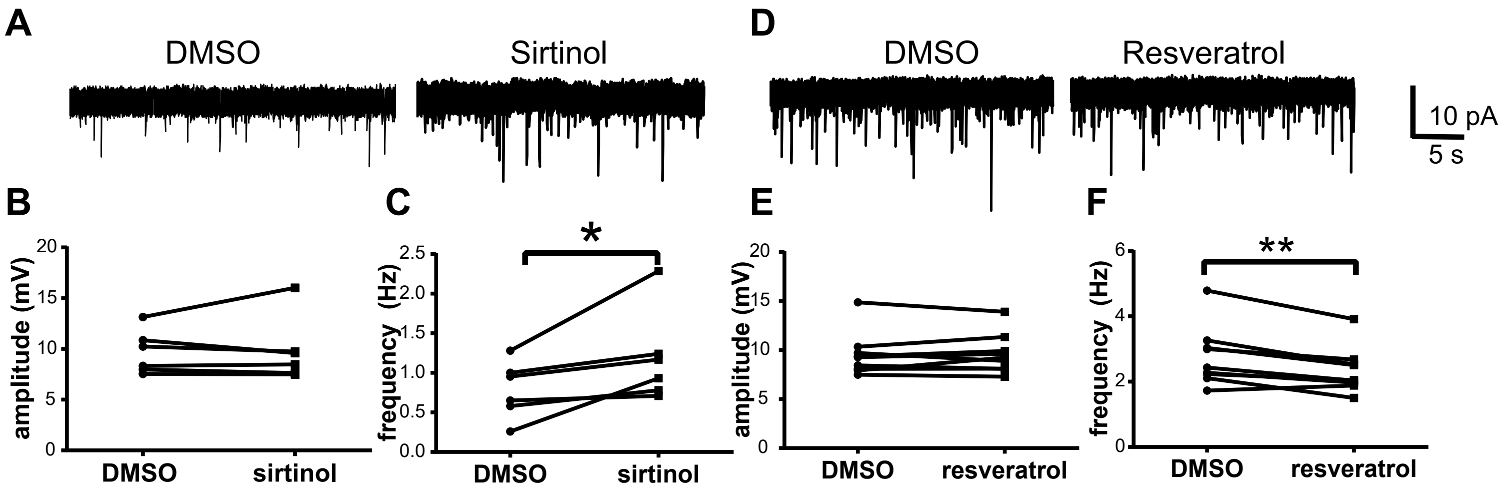

Supplementary Figure 2. Sirtinol and Resveratrol regulated sEPSC frequency in the dentate gyrus granule cells in the same way as SIRT1 inhibitor IV and SIRT1 activator 3, respectively. **A**. top traces are sEPSC recording from the same cell before and after application of 30 μM Sirtinol at a holding potential of -70mV. **B.** Sirtinol application significantly increased sEPSC frequency (paired t-test, t(5)=2.69, P=0.04) but no significant effect on sEPSC amplitude (**C**, paired t-test, t(5)=0.25, P=0.81). **D**. top traces are sEPSC recording from the same cell before and after application of 200 μM resveratrol at a holding potential of -70mV. **E.** Resveratrol application significantly decreased sEPSC frequency (paired t-test, t(8)=4.01, P<0.01) but no significant effect on sEPSC amplitude (**F**, paired t-test, t(8)=0.37, P=0.72).

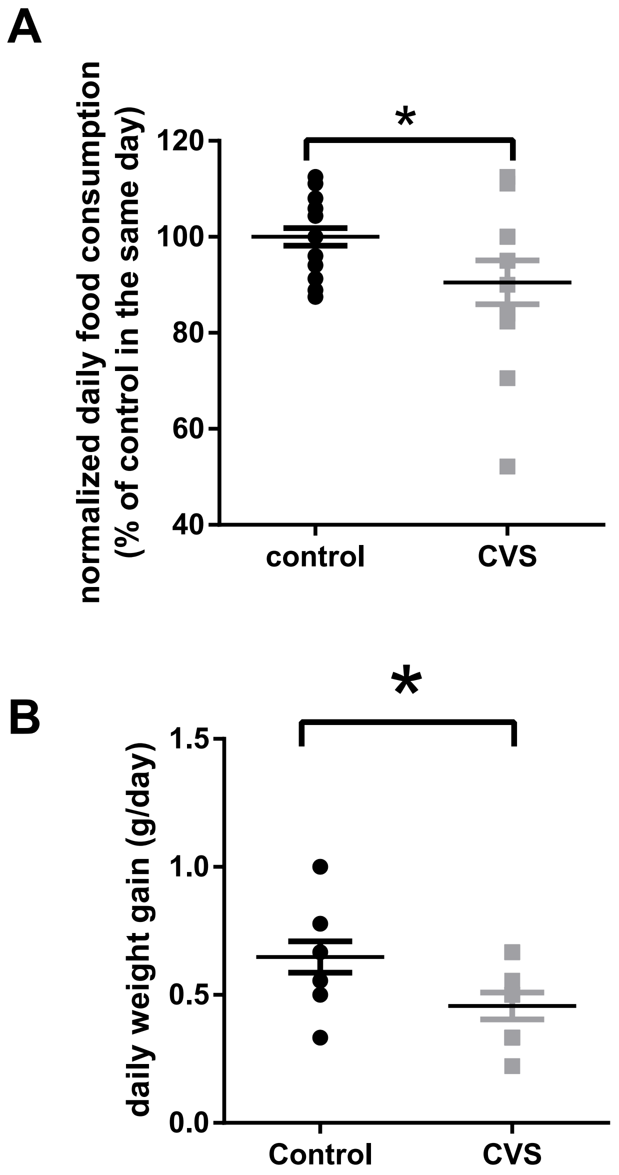

Supplementary Figure 3. CVS-treated mice consumed significant less food and gained significant less weight compared to control mice. Daily food consumption **(A)** and daily weight gain **(B)** were monitored every day to assess the CVS conditions of mice. These results (reduced food consumption and weight gain) demonstrate the effectiveness of our CVS protocols.

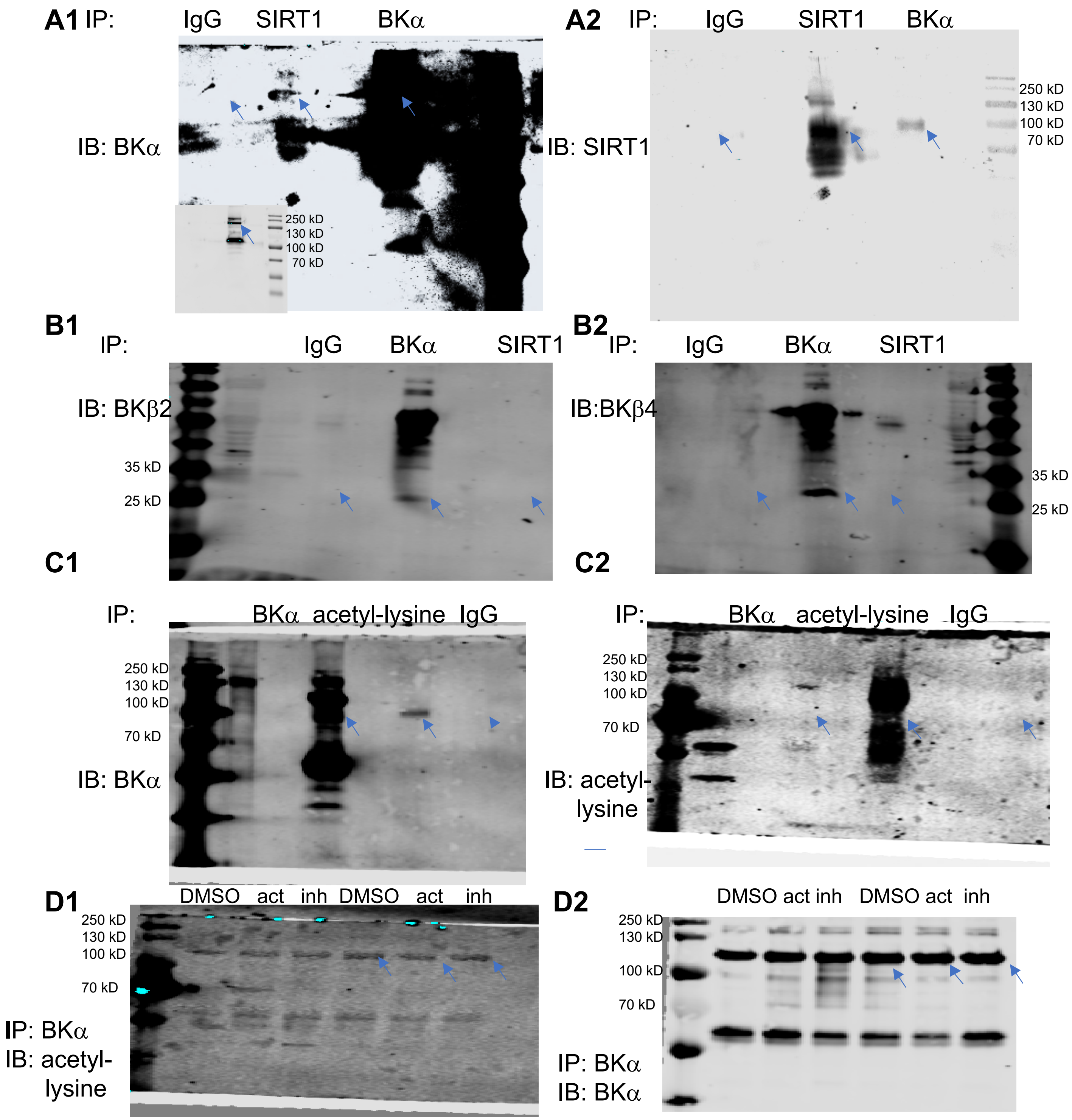

Supplemental Figure 4: Full Western blots for figures shown if figure 4. The panel letter in supplemental figure correlates to the panel letter in figure 4. Inset in A1 shows a shorter exposure to reveal the marker. The blue arrows shown here indicate the bands shown in the figure 4.

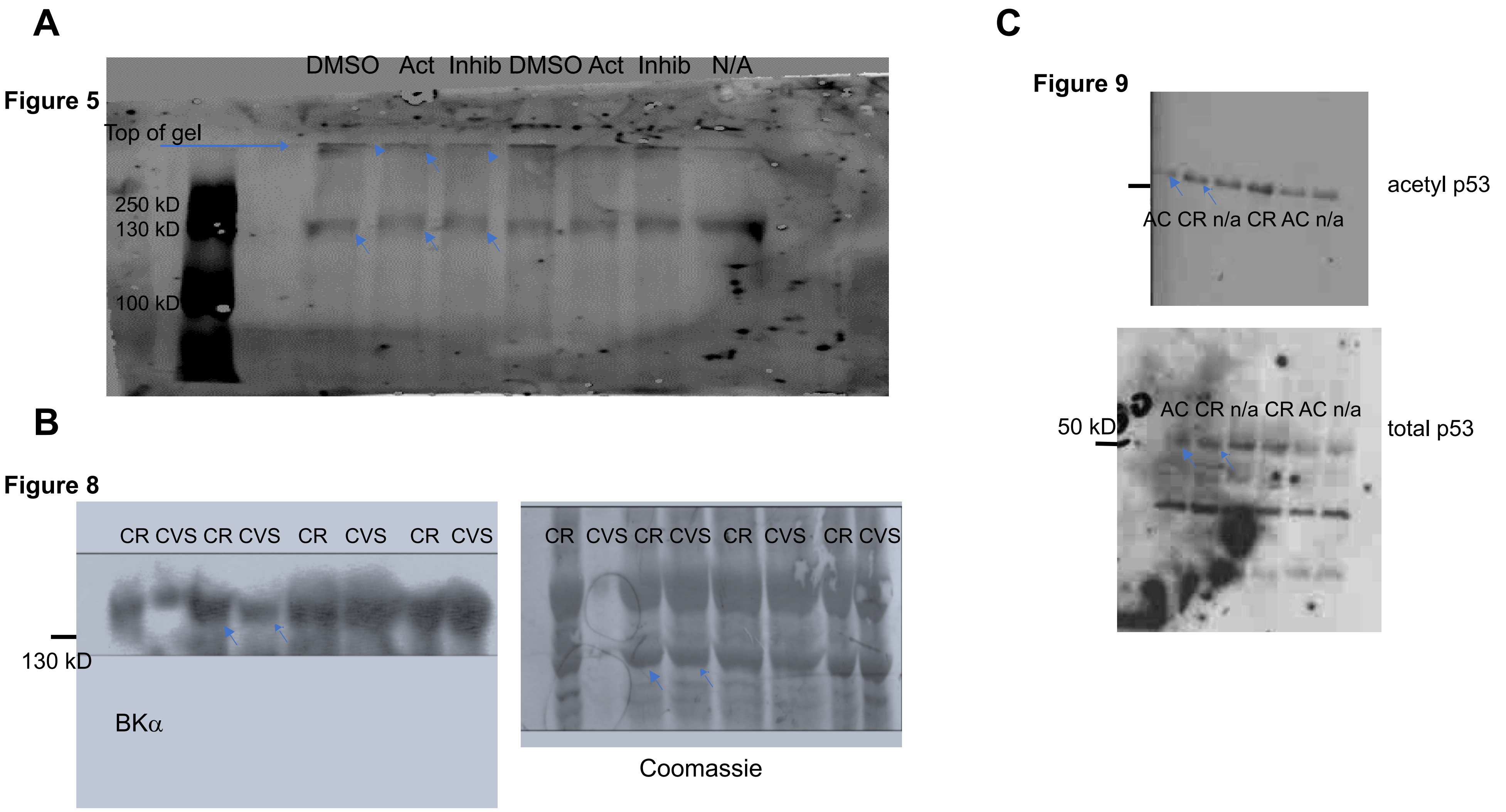

Supplemental figure 5. Entire Western blots for figures shown in figure 5 (A), figure 8 (B) and figure 9 (C). The blue arrows show the representative bands shown the figures.

		V_m (mV)	R_{input}(MΩ)	sEPSC amplitude (pA)	sEPSC decay time constant (ms)	mEPSC amplitude (pA)	mEPSC decay time constant (ms)	AP rise time (ms)
**SIRT1 inhibitor IV (1μM)**	**control**	-73.34±0.87	589.67±41.15	8.78±0.58	4.47±0.39	8.41±0.59	5.35±0.22	2.03 ±0.36
	**drug**	-73.65±0.84	548.67±60.80	8.58±0.51	4.68±0.36	8.20±0.41	4.96±0.27	1.88 ±0.23
**SIRT1 activator 3 (50μM)**	**Control**	-72.1±2.00	498.36±40.92	9.35±0.51	4.13±0.19	7.53±0.28	4.15±0.16	2.13 ±0.25
	**drug**	-69.34±2.76	513.09±38.79	8.80±0.61	4.15±0.19	7.43±0.28	4.37±0.16	2.01 ±0.222
**CVS+ SIRT1 inhibitor IV (1μM)**	**CVS**	-71.64±2.43	487.33±40.07	7.92±0.33	4.48±0.19	7.34±0.25	4.08±0.28	2.83 ±0.24
	**CVS+drug**	-72.84±3.31	496.45±29.06	7.71±0.34	4.55±0.28	7.27±0.28	4.12±0.29	2.72± 0.22
**CVS+ SIRT1 activator 3 (50μM)**	**CVS**	-70.52±2.40	554.14±47.65	8.11±0.45	4.18±0.14	7.56±0.26	4.09±0.23	2.26 ±0.21
	**CVS+drug**	-73.00±2.34	569.52±42.23	7.65±0.36	4.15±0.17	7.28±0.30	4.05±0.25	2.23 ±1.96

Supplementary Table 1. Properties of dentate gyrus granule cells recorded before and after drug application in control and CVS mice. No significance found; V_m: resting potentials; R_{input}: input resistance tested at the voltage of -65mV; AP: action potentials; mean±SEM shown.
